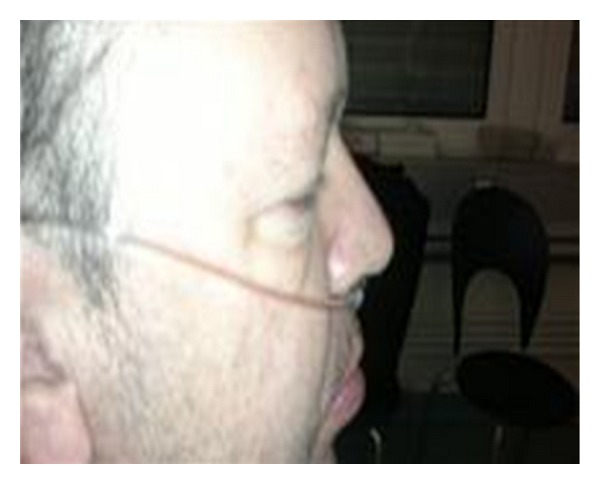# Acquired Form of Angioedema of the Head and Neck Related to a Deficiency in C1-Inhibitor: A Case Report with a Review of the Literature

**DOI:** 10.1155/2012/405824

**Published:** 2012-12-10

**Authors:** Bassel Hallak, Propser Konu, Florian Lang, Christian Simon, Philippe Monnier

**Affiliations:** ^1^Department of Otorhinolaryngology, University Hospital CHUV, Rue du Bugnon 46, 1011 Lausanne, Switzerland; ^2^Department of Otorhinolaryngology, Cantonal Hospital Fribourg, Fribourg, Switzerland

## Abstract

Angioedema related to a deficiency in the C1-inhibitor protein is characterized by its lack of response to therapies including antihistamine, steroids, and epinephrine. In the case of laryngeal edema, mortality rate is approximately 30 percent. The first case of the acquired form of angioedema related to a deficiency in C1-inhibitor was published in 1972. In our paper, we present a case of an acquired form of angioedema of the oropharyngeal region secondary to the simultaneous occurrence of two causative factors: neutralization of C1-inhibitor by an autoantibody and the use of an angiotensin convertin enzyme inhibitor.

## 1. Introduction

Angioedema results from a loss of vascular integrity that allows fluid to escape into soft tissues. Exposure of the vasculature to inflammatory mediators causes dilatation and increased permeability of capillaries and venules.

The known causes of angioedema can be subdivided into three groups depending upon the underlying mechanism: mast cell mediated, bradykinine mediated, and unknown etiology.

The angioedema related to a deficiency in protein C1-inhibitor has bradykinine as a mediator. This deficiency can be hereditary or acquired. Edema is characterized by its lack of response to therapies including antihistamine, steroids, and epinephrine [1]. We present a case of angioedema of the head and neck related to an acquired deficiency in the protein C1-inhibitor.

## 2. Case Report

**Figure 1:**
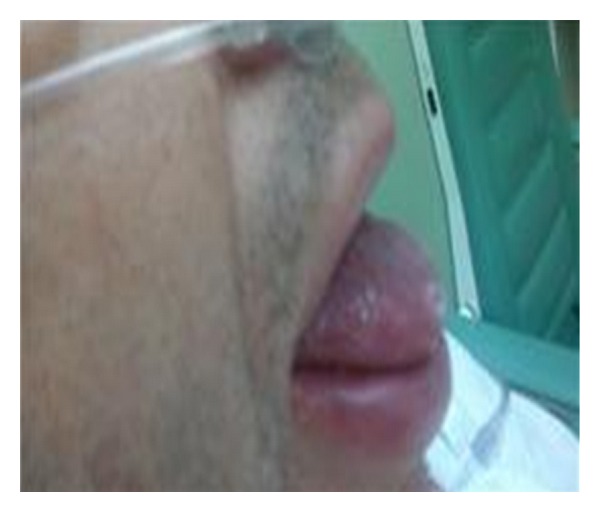


A 53-year-old man presented to the emergency department with a severe edema of the lower lip and tongue (Figure 1). The edema developed suddenly without any obvious history of trauma or foreign body ingestion. The clinical investigation did not show any history of allergic disorders. There was no obvious history of edema. The patient was in good health and was only known for arterial hypertension treated by an angiotensin converting enzyme inhibitor. He had been using this single medication for a few months. He did not have any dyspnea and suffered only from swallowing difficulties associated with modification of the voice. The clinical examination did not show any other systemic disease, or any edema at the level of the larynx.

A classic treatment of antihistamines, epinephrine, and steroids, 250 mg bid by intravenous administration was introduced. No clinical response was noted nor any decrease in the size of the edema. Progressively, respiratory distress related to the extension of the edema to the tongue base became conspicuous.

A dose of 25 U/kg of C1-INH concentrate was then given intravenously.

The edema disappeared completely within 35 minutes after administration of the CI-INH concentrate (Figure 2). 

The patient was followed up and no recurrence of the edema was noted.

The biologic findings showed a level of C3 at 1.01 g/L (normal levels: 0.75–1.40 g/L), and of C4 at 0.31 (normal 0.15–0.35 g/L). The weight continent of C1-INH was at 0.30 g/mL (normal between 0.21–0.39 g/L), and the functional level was at 61 ± 10% (normal between 70–130%).

We noted that the level of the anti-C1-INH autoantibodies was positive and elevated to more than 23 U/mL (normal value ≤ 20 U/mL).

## 3. Discussion

The C1-inhibitor (C1-INH) is an acute-phase reactant protein and is the primary inhibitor of the classical complement pathway as well as of the coagulation (contact system), fibrinolytic, and kinin-generation pathways [2, 3].

C1-INH inhibits the following plasma components of these pathways:

- Hageman factor (factor XII),
- clotting factor XI and XIIa,
- plasma kallik.

Hereditary angioedema (HAE) is a rare autosomal dominant genetic disorder resulting from an inherited deficiency or dysfunction of the C1-INH. The prevalence of HAE is estimated at 1 individual per 50,000, with reported ranges of 1 : 10,000 to 1 : 150,000 [4, 5]. There are no known differences in prevalence among ethnic groups [6]. Men and women are affected equally. Two subtypes of HAE have been defined.

Type I HAE accounts for 85% of the cases and is characterized by low levels of functional C1-INH. The levels can occasionally drop to 30–50% of normal values in most patients [7, 8].

Type II HAE results from the presence of a dysfunctional C1-INH, which is present in normal or elevated amounts [8].

The gene for C1-INH maps to the long arm of chromosome 11. More than 100 mutations have been reported in unrelated patients with HAE types I and II [9]. 

Patients with hereditary angioedema typically present in late childhood or early adolescence with angioedema following trauma, infection, dental procedures, or emotional stress, with an increasing frequency and severity of episodes with puberty, menses, and ovulation. These patients are otherwise healthy.

Acquired angioedema (AAE) is most common in older patients (>50 years), and most patients have associated concomitant diseases. It can be divided into three subtypes.

- Type I is due to an excessive consumption of C1-INH induced by hyperactivation of the classic complement pathways with immune circulate complexes (lymphoproliferative syndrome, autoimmune diseases).
- Type II is due to a neutralization of C1-INH by autoantibodies.
- Type III is due to angiotensin converting enzyme inhibitors (ACE inhibitors). The angioedema occurs in 0.1% to 0.7% of patients treated with this medication [10, 11]. The ACE inhibitors account for 20% to 30% of all angioedema cases presenting to emergency departments.

The attacks of the angioedema most often affect three anatomical locations: the skin (cutaneous attack), gastrointestinal tract (gastrointestinal attacks), and upper airway (laryngeal/pharyngeal attacks).

Symptoms with both forms of C1-INH deficiency can range in severity from a minor inconvenience to a life-threatening laryngeal edema by fatal asphyxiation with a mortality rate of approximately 30% [12, 13]. Laryngeal edema occurs in approximately one-half of all patients over their lifetime, although only a few experience recurrent episodes.

Laryngeal attacks account for less than 1% of all angioedema episodes, and they are less common in patients over age of 45 years [14]. The laryngeal swelling usually develops over hours, with a reported mean of 7 hours.

Gastrointestinal attacks are experienced by a majority of patients with hereditary angioedema and present as varying degrees of gastrointestinal colic, nausea, vomiting, and/or diarrhea, which result from bowel wall edema. Because of the clinical similarities between bowel attacks of angioedema and true surgical emergencies, as many as one-third of the patients with undiagnosed hereditary angioedema may undergo unwarranted abdominal surgery [15].

The impact of hormonal fluctuations (including pregnancy) in women with hereditary angioedema is variable [16, 17].

The mechanism of angioedema induced by angiotensin converting enzyme inhibitors (ACE inhibitors) is a class effect that is directly related to the mechanism of action. Thus, the ACE inhibitors have the effect of decreasing angiotensin II and increasing bradykinin production. One report described a patient with ACE inhibitor-induced angioedema whose bradykinin levels rose acutely during the episode and normalized after it [11].

The C1-INH deficiency-induced angioedema is distinguished by its lack of response to therapies for other types of angioedema, including antihistamines, steroids, and epinephrine.

The effective therapy is to replace the inhibitor (administration of C1-INH concentrate or fresh frozen plasma) in case of severe edema, especially of the respiratory tract. Increasing the hepatic synthesis of C1-INH, the anti-fibrinolytic agents, or blocking bradykinin formation or its receptor engagement should be attempted in cases of moderate or recurrent episodes.

Prior to the introduction of effective therapies for HAE, up to one-third of patients died of asphyxiation [17]. However, despite effective therapies, deaths secondary to laryngeal attacks still occur with some regularity, although data are limited. A series of Austrian, Swiss, and German patients published in 2004 cited a mortality rate as high as 13% [1].

In our patient, the mechanism of edema formation was related to two factors: the use of an ACE inhibitor and the neutralization of C1-INH by an autoantibody anti-C1-INH. This result is compatible with an acquired form of angioedema related to a deficiency in C1-INH (the first case was published in 1972). Only 50 cases of the acquired form of angioedema have been published in the literature. Our case is the first in which the two causative factors could be identified in the same patient.

## Figures and Tables

**Figure 2:**